# Biostimulant Effects of *Trichoderma asperellum* in Hydroponic Spinach Production

**DOI:** 10.3390/life15030428

**Published:** 2025-03-08

**Authors:** Jared Hernández-Huerta, Brenda I. Guerrero, Angélica Anahí Acevedo-Barrera, Martha Irma Balandrán-Valladares, Rosa María Yañez-Muñoz, Ndahita De Dios-Avila, Aldo Gutiérrez-Chávez

**Affiliations:** Department of Agrotechnological Sciences, Autonomous University of Chihuahua, Campus 1, Av. Pascual Orozco S/N, Chihuahua 31350, Mexico; brguerrero@uach.mx (B.I.G.); aacevedo@uach.mx (A.A.A.-B.); mbalandran@uach.mx (M.I.B.-V.); myanez@uach.mx (R.M.Y.-M.); ndedios@uach.mx (N.D.D.-A.)

**Keywords:** hydroponic, biostimulants, spinach, fungi

## Abstract

Using beneficial microorganisms as biostimulants is a promising strategy to enhance crop growth and productivity in hydroponic systems. *Trichoderma asperellum* has demonstrated plant growth-promoting effects in soil cultivation; however, its efficacy in hydroponic Deep-Water Culture (DWC) systems remains underexplored. This research evaluated the effect of *T. asperellum* strains TaMFP1 and TaMFP2 on the growth, nutrient uptake, and visual quality of hydroponically grown spinach cv. Stella Plus F-1 (*Spinacia oleracea* L.). A randomized complete design was used, comparing inoculated plants with a control and a commercial *Trichoderma harzianum* product. After 28 days, the results showed that *T. asperellum* significantly increased plant height (23.1%), collar diameter (21.8%), root length (39.6%), leaf area (22.0%), number of leaves (18.05), and fresh biomass accumulation (23.5%) compared to non-inoculated plants. Furthermore, inoculation with TaMFP1 improved spinach yield by 34.5%, while nutrient analysis indicated enhanced phosphorus and calcium uptake. No significant changes were observed in photosynthetic pigment concentrations, and the visual quality of the harvested spinach was unaffected. These findings highlight the potential of *T. asperellum* as a sustainable biostimulant in hydroponic spinach production. These results contribute to developing beneficial microorganism-based strategies to enhance the sustainability of hydroponic agriculture.

## 1. Introduction

The use of beneficial microorganisms as biostimulants has gained relevance in modern agriculture due to their ability to enhance plant growth, optimize nutrient absorption, and increase resistance to abiotic and biotic stress [1,2,3,4]. Among these microorganisms, the *Trichoderma* genus stands out for its versatility and efficacy in promoting plant growth [5]. Its application in crops has demonstrated benefits in both productivity and sustainability [6,7,8]. Within the *Trichoderma* genus, *Trichoderma asperellum* has been studied for its biostimulant capacity [9,10]. This fungus establishes beneficial interactions with plant roots, promoting their development and facilitating substrate exploration [5]. Additionally, it produces bioactive compounds that stimulate cell division and the synthesis of phytohormones essential for plant growth [6,10]. It also enhances the availability of nutrients such as phosphorus and iron by solubilizing them in the growing medium [11]. However, despite these benefits, most studies on *T. asperellum* have been conducted in soil-based systems, and its behavior in hydroponic environments remains insufficiently explored. Understanding its potential in soilless cultivation is crucial for expanding its application in modern agriculture.

While the use of *T. asperellum* in agricultural soils is well documented, its application in hydroponic systems remains an area of exploration. Some studies have shown that *T. asperellum* strains NST-099 and CB-Pin-01 in hydroponic systems inhibit pathogens such as *Cercospora lactucae-sativae* and *Pythium aphanidermatum*, while also improving lettuce (*Lactuca sativum* L.) growth parameters, including plant height, leaf number, and biomass [12,13]. Additionally, *T. asperellum* strains TaMFP1 and TaMFP2, inoculated in hydroponic lettuce, have promoted vegetative growth parameters, such as plant height, root length, biomass, and leaf number, without affecting visual quality [14]. Likewise, the application of TaMFP1 in hydroponically grown pea microgreens (*Pisum sativum* L.) enhanced their growth, while its combination with *Bacillus thuringiensis* Bt24 reduced nitrate levels [15]. Moreover, *T. asperellum* T-203 has been shown to induce systemic resistance in cucumber (*Cucumis sativum* L.) against bacterial pathogens such as *Pseudomonas syringae*, reducing disease symptoms through the production of phytoalexins [16]. Despite these promising findings, the variability in microbial interactions within hydroponic environments suggests that further investigation is needed to determine the consistency of *T. asperellum*’s biostimulant effects across different crops and cultivation systems.

Hydroponic systems have distinct conditions, such as the continuous availability of nutrients and the absence of organic matter, which may alter the interactions between roots and beneficial microorganisms [17,18,19,20]. Assessing the behavior of *T. asperellum* in these systems is key to determining its effectiveness as a biostimulant in soilless cultivation. One of the most widely used hydroponic systems for leafy vegetable production is the Deep-Water Culture technique [21]. This method is recognized for its water-use efficiency and stability in plant nutrition [22]. However, factors such as dissolved oxygen availability and microbial competition in the nutrient solution can influence plant growth [23]. Given these factors, it is essential to evaluate whether introducing *T. asperellum* in DWC can enhance plant performance, particularly regarding growth and quality.

In this context, spinach (*Spinacia oleracea* L.) is a high-value commercial and nutritional crop whose hydroponic production has proven to be more efficient and of better quality compared to conventional cultivation [24,25,26]. However, in hydroponic systems, the absence of beneficial microbiota like those found in soil can limit plant growth and resistance [27,28]. It has been reported that this reduced microbial biodiversity can decrease nutrient absorption and increase susceptibility to diseases [20,29,30]. Given the economic and nutritional importance of spinach, identifying bio-based strategies that enhance its hydroponic production is a priority for sustainable agriculture.

This study presents a novel approach by evaluating the biostimulant effect of *T. asperellum* in hydroponic spinach production using a Deep-Water Culture system. While previous research has focused on its application in soil-based systems or other hydroponic crops, limited information is available regarding its impact on hydroponically grown spinach. This research contributes original findings by assessing its influence on morphological, nutritional, and visual quality parameters, providing insights into the potential of *T. asperellum* to optimize soilless spinach cultivation. Therefore, this research aimed to evaluate the effect of *T. asperellum* as a biostimulant on the growth and development of spinach grown in a Deep-Water Culture system. Morphological, nutritional, and visual quality parameters of the plant were analyzed to determine the impact of this microorganism on the efficiency of the production system. Under the hypothesis that applying *T. asperellum* in hydroponic spinach cultivation would enhance plant growth and quality, thereby increasing the overall efficiency of the production system, this research aims to elucidate its role in this type of cultivation. The results will contribute to developing sustainable cropping strategies that reduce dependency on chemical inputs while maintaining or improving crop yield and quality.

## 2. Materials and Methods

### 2.1. Localization

The research was carried out at the Laboratory of Applied Microbiology, Phytopathology, and Postharvest Physiology (LAMPPP) and in the tunnel-type greenhouse (6 m in height, 20 m in length, and 15 m in width) located at the Faculty of Agrotechnological Sciences of the Autonomous University of Chihuahua (UACH) in Chihuahua, Mexico (28°39′24″ N, 106°05′12″ W). The experiment was conducted from 10 October to 22 November 2024, with 15 days for the seedling production stage and 28 days for the hydroponic cultivation trial in the greenhouse.

### 2.2. Microorganisms

The strains of *Trichoderma asperellum* TaMFP1 (GenBank: PQ786128) and *T. asperellum* TaMFP2 (GenBank: PQ786128), evaluated as biostimulants, were obtained from the LAMPPP at the Faculty of Agrotechnological Sciences, UACH. These strains have demonstrated biostimulant activity in chili pepper (*Capsicum annuum* L.), lettuce (*Lactuca sativa* L.), and pea (*Pisum sativum* L.) in studies conducted at the LAMPPP, UACH [14,15]. *Trichoderma harzianum* (Trichospore^®^, Bioproducos Laguneros, Torreón, COAH, Mexico) was used as a positive control. This beneficial fungus is employed as a biological control agent against other fungi, promoting plant growth by releasing nutrients in forms available to plants and producing growth-regulating hormones. It should be noted that this information is based on the manufacturer’s claims (www.bioproductoslaguneros.com (accessed on 25 January 2025)).

### 2.3. Preparation of Inoculum

The evaluated *Trichoderma* strains were cultivated on Potato Dextrose Agar (PDA; BD Difco Laboratories, Sparks, Maryland, MD, USA) in Petri dishes under controlled conditions at 28 °C for seven days using an incubator (LAB-Line Imperial III, Fisher Scientific, Dallas, TX, USA). After the incubation period, conidia were collected by carefully scraping the fungal mycelium with a sterile spatula. The harvested conidia were then suspended in 10 mL of sterile water and mixed for 2 min using a vortex mixer set to speed 4 (Daigger Vortex-Genie2; Scientific Industries Inc., Bohemia, NY, USA). The conidia suspension was filtered through a syringe fitted with a glass fiber filter to obtain a purified conidial solution. Conidial concentration was adjusted to 1 × 10^6^ conidia/mL (Neubauer counting chamber, Weber Scientific International Ltd., Teddington, UK) with 0.03% (*w*/*v*) xanthan gum (Sigma-Aldrich Quimica, Toluca, MEX, Mexico) [31].

### 2.4. Seedling Production

The spinach (*Spinacia oleracea* L.) selected for this study corresponded to the cultivar Stella Plus F-1 (kristenSeed^®^, Guadalajara, JAL, Mexico), an Asian-type spinach known for its adaptability. It is resistant to *Peronospora farinosa f.* sp. *spinaciae* and *Peronospora efussa*. Additionally, it exhibits resistance to dry and humid heat, making it an excellent choice for commercial cultivation throughout the year (www.kristenseed.com.mx (accessed on 25 January 2025)).

Seeds of spinach were sown in germination trays with drainage holes (31 cm × 25 cm × 4.5 cm: Eassty^®^, B0BQ3KWRZ1, Philadelphia, PA, USA) with sphagnum peat moss substrate (Premier^®^, Premier Tech Horticulture, Rivière-du-Loup, QC, Canada). Then, they were placed in the greenhouse, where they remained until they developed two true leaves before being transplanted into the hydroponic system. The seeds were irrigated with water every four days, and once germination occurred, they received Steiner nutrient solution (Inverfams^®^, Querétaro, QE, México). The solution contained the following concentrations (ppm): 126 NO^3−^, 42 NH^4+^, 31 PO_4_^3−^, 274 K^+^, 181 Ca^2+^, 48.6 Mg^2+^, 112 SO_4_^2−^, 1.3 Fe (as ethylenediaminetetraacetic acid, EDTA), 0.8 Mn-EDTA, 0.3 Zn-EDTA, 0.06 Cu-EDTA, 0.4 B, and 0.06 Mo. The pH level was adjusted to remain within the range of 6.0–6.2, while the electrical conductivity (EC) was maintained at 1.5 mS/cm [32].

### 2.5. Trial Establishment

Spinach seedlings, 15 days old, were root-inoculated with 3 mL of *Trichoderma* conidial suspension (1 × 10^6^ conidia mL^−1^) by spraying using an atomizer (JR-24/410, MultiPlastic^®^, Tlajomulco de Zuñiga, JAL, Mexico). After inoculation, the seedlings were placed individually in a Deep-Water Culture (DWC) system. The inoculation was repeated three times at 7-day intervals.

The DWC system consisted of rectangular, straight-wall polypropylene containers with a capacity of 14.0 gallons (53 L) and dimensions of 15 inches in width (W) by 24 inches in length (L) by 9 inches in height (H) (Interstack, Europlast^®^, Queretaro, QRO, Mexico). The cultivation system consisted of polystyrene plates with a thickness of 1 inch and dimensions of 15 inches W by 24 inches L, positioned within each containment unit. Each plate featured eight evenly spaced holes, each with a diameter of 5 cm and a 10 cm separation, designed for seedling placement.

The oxygenation of the DWC system was maintained using an air pump with a capacity of 65 L/min (K003, Raoping Xingcheng Electromechanical Aquarium Supplies, Guangdong, China), which was regulated by a manual timer programmed to activate for 15 min every 5 h during the experiment (B012890E7Y, Volteck^®^, Ciudad de México, Mexico). The aeration setup included an adjustable dripper with a flow range of 0–70 L/h (B07BTDDDJKJ, Zerodis, Hydroenviroment^®^, Tlalnepantla, Mexico), which was linked to the air pump via 4/7 mm micro tubing (Hydroenviroment^®^, Tlalnepantla, Mexico).

A Steiner nutrient solution served as the primary nutrient source, ensuring a pH stability between 6.0 and 6.2, while the EC was maintained at 1.5 mS/cm [32]. The study took place in a greenhouse setting, where environmental conditions included a minimum temperature of 15.8 °C, a maximum of 25.3 °C, and an average relative humidity of 71.2%.

### 2.6. Plant Analysis

#### 2.6.1. Morphological Parameters

The morphological variables of spinach plants were evaluated 28 days after the first inoculation with the fungus. Four plants per replication were measured, with four replicates per treatment. Measurements included plant height (from the base to the highest leaf), stem diameter, root length, and number of leaves per plant, and leaf area was determined using the Canopeo^®^ phone app [33], allowing for green canopy coverage estimation through digital imaging.

The fresh and dry biomass of shoots, roots, and the entire plant were measured separately. Fresh biomass was weighed immediately after harvest. The dry biomass was determined after being dried in a forced-air convection oven (SMO3, Shel Lab^®^, Cornelius, OR, USA) at 75 °C until a constant weight. The fresh and dry biomass was measured with an analytical balance (XT-220A, Precisa Instruments^®^, Zurich, Switzerland), with results expressed in grams per plant (g plant^−1^).

The shoot–root ratio was calculated using the formula proposed by Ericsson [34] (1):

(1) Shoot:root ratio=Dry weigh of shoot (g)/Dry root biomass (g)

The leaf area ratio (LAR) was calculated using the formula suggested by Parwada et al. [35] (2):

(2) $$\mathrm{LAR}=\mathrm{Total}\;\mathrm{leaf}\;\mathrm{area}\;\mathrm{of}\;\mathrm{plant}\;({\mathrm{cm}}^{2})/\mathrm{Plant}\;\mathrm{dry}\;\mathrm{weight}\;(g)$$

The specific root length (SRL) was calculated using the formula suggested by Fujita et al. [36] (3):

(3) SRL =Root length (m)/ Root dry weight (g)

#### 2.6.2. Photosynthetic Pigments

The quantification of photosynthetic pigments, including chlorophyll *a*, chlorophyll *b*, and carotenoids, was conducted following the methodology described by Lichtenthaler and Wellburn [37], 28 days after the first fungal inoculation. Two composite samples were taken from each replicate per treatment, using leaf blades from the middle part of two leaves per plant. Pigments were extracted from 0.1 g of fresh leaf samples (W) by homogenization in 4 mL of 80% acetone (Sigma-Aldrich, St. Louis, MI, USA) (V). The extract was then centrifuged at 3000 rpm for 5 min. The absorbance supernatant was measured at 663, 645, and 470 nm using a UV–visible spectrophotometer (Evolution 60S, Thermo Scientific^®^, Madison, WI, USA). The pigment concentrations were calculated using the following Formulas (4)–(6):

(4) $$\mathrm{Chlorophyll}\;a\;(\mathrm{mg}g^{-1}\mathrm{FW})=\left(12.21\;\times A_{663}-2.81\;\times A_{645}\right)\;\times \;V/(1000\;\times \;W)$$

(5) $$\mathrm{Chlorophyll}b\left(\mathrm{mg}g^{-1}\mathrm{FW}\right)=\left(20.13\;\times A_{645}-5.03\;\times A_{663}\right)\;\times \;V/(1000\;\times \;W)$$

(6) $$\mathrm{Carotenoids}\;\left(\mathrm{mg}\;g^{-1}\mathrm{FW}\right)=\left\lfloor \frac{\left(1000\;\times A_{470}-3.27\;\times {\mathrm{Chl}}_{a}-104\;\times {\mathrm{Chl}}_{b}\right)}{229}\right\rfloor \times \;V/(1000\;\times \;W)$$

where FW = fresh weight of the samples (g).

#### 2.6.3. Foliar Nutrient Content

The nutrient composition of spinach leaves was evaluated following a drying process in a forced-air convection oven (SMO3, Shel Lab^®^, Cornelius, OR, USA) at 60 °C for 96 h. Once dried, the plant material was ground to a fine powder with a particle size of 1 mm using a sieve (Thomas Scientific^®^, Swedesboro, NJ, USA). The analysis considered all plants from each replication, with four plants per replication. The total nitrogen percentage was measured through the Kjeldahl method, employing a Novathec^®^ Digester (San Pedro Tlaquepaque, JAL, Mexico) and a Micro Kjeldahl Labconco^®^ Rapid Distillation Unit (Kansas City, MO, USA) [38]. To determine the concentrations of Cu, Fe, Mn, and Zn, a tri-acid digestion was performed using a mixture of HNO_3_, HClO_4_, and H_2_SO_4_ in a ratio of 10:1:0.25, with 0.1 g of dried plant material [39]. These elements were quantified through atomic absorption spectrometry (Perkin Elmer Analyst 100, Waltham, MA, USA) and the results were expressed in ppm based on dry plant weight. The contents of Ca, Mg, and K were analyzed from previously digested samples, which were diluted to a 1% solution using deionized water. Their concentrations were determined through atomic spectrometry and reported as percentages. Lastly, total phosphorus content was assessed via the vanadate–molybdate method, measuring absorbance at 410 nm using a UV–visible spectrophotometer [40].

### 2.7. Crop Yield

The yield of the spinach was calculated using the formula suggested by Moreira et al. [41] (7):

(7) $$\mathrm{Yield}\;\left(\mathrm{Kg}\;m^{2}\right)=\mathrm{Shoot}\;\mathrm{fresh}\;\mathrm{weight}\;\left(\mathrm{kg}\right)\;\times \;\mathrm{Plant}\;\mathrm{population}\;\mathrm{per}\;m^{2}$$

A density of 34 plants per m^2^ was considered for the calculation of yield.

### 2.8. Visual Quality

The quality of the spinach was evaluated using the scale suggested by Bergquist et al. [42]. For the analysis, all plants per replication were considered, with four plants per replication (Table 1):

### 2.9. Experimental Design

The experiment was arranged following a completely randomized design, incorporating four different treatments with four replications each. Every replication consisted of four individual plants. The treatments were as follows: (1) plants inoculated with *T. asperellum* strain, (2) plants inoculated with *Trichoderma* strain TaMFP2, (3) plants inoculated with *T. harzianum* (Th-CP), and (4) a control group with non-inoculated plants.

### 2.10. Statistical Analysis

To assess the growth response of spinach plants to *Trichoderma* inoculation in the DWC system, data were first tested for normality using the Shapiro–Wilk test and for homogeneity of variances using Levene’s test. Depending on these preliminary results, the data were analyzed using one-way analysis of variance (ANOVA) followed by Tukey’s post hoc test, Welch’s ANOVA coupled with the Games–Howell test, or the Kruskal–Wallis test followed by the Conover–Iman post hoc test (*p* < 0.05). The visual quality of the spinach plants was examined using contingency tables and the Chi-square test [43].

A principal component analysis (PCA) was conducted to identify variations among treatments. Prior to performing the PCA, Bartlett’s test of sphericity was applied to verify the appropriateness of the dataset (*p* < 0.01). Additionally, the dataset’s suitability for PCA was assessed using the Kaiser–Meyer–Olkin (KMO) test, with values exceeding 0.60 considered acceptable [44]. All statistical analyses were carried out using JAMOVI 2.5.2.0 software (The Jamovi Project, 2024), which can be accessed at https://www.jamovi.org (accessed on 12 January 2025).

## 3. Results

### 3.1. Trichoderma as a Growth Promoter of Spinach

The findings of this research indicate that the growth of spinach cv. Stella F-1 in a DWC system was notably enhanced through root inoculation with *Trichoderma* spp. While the overall morphological traits of the inoculated spinach plants resembled those of their non-inoculated counterparts, significant differences were observed in the vegetative parameters (*p* < 0.05) (Table 2 and Table 3).

All fungal treatments significantly enhanced the plant height, collar diameter, root length, and leaf area by 23.10%, 21.67%, 39.62%, and 22.0%, respectively, compared to the control. Additionally, the number of leaves per plant increased by 18.05% with the TaMFP1 and TaMFP2 treatments compared to the non-inoculated plants. No significant differences were observed in the LAR among treatments, with an average of 254.90 cm^2^/g of biomass. Regarding SRL, a significant enhancement was observed only with the TaMFP2 treatment, showing a 32.98% increase compared to the control (Table 2).

Regarding fresh biomass, the highest increase in leaf biomass was observed with the TaMFP2 treatment, showing a 28.93% improvement compared to the control. Likewise, root fresh weight increased by 34.53% with the TaMFP1 treatment. On the other hand, both TaMFP1 and TaMFP2 improved total fresh weight by 23.45% compared to the non-inoculated plants (Table 3).

In the case of dry biomass, all fungal treatments resulted in the same increase in leaf dry weight, with a 27.38% improvement compared to non-inoculated plants. Root dry weight improved by 26.32% with the TaMFP1 treatment. Total dry weight increased by 26.14% with all fungal treatments. No significant differences were observed in the shoot–root ratio among treatments, with an average of 8.95 (Table 3).

### 3.2. Photosynthetic Pigments

The photosynthetic pigments content was unaffected by *Trichoderma* treatments, with values of 1.25 mg g^−1^ FW of chlorophyll a, 0.615 mg g^−1^ FW of chlorophyll b, and 0.541 mg g^−1^ FW of carotenoids (Table 4).

### 3.3. Nutritional Content of Spinach

Significant differences (*p* < 0.05) in macro-and micronutrient levels were observed in spinach treated with *Trichoderma* (Table 5). The nitrogen and magnesium content increased by 18.78% and 4.87%, respectively, only with the Th-CP treatment compared to the control. The phosphorus content was significantly higher with the TaMFP1 treatment, achieving a 13.39% increase compared to non-inoculated plants. Calcium content also increased with fungal treatments, with the highest increase obtained with Th-CP by 32.59%, followed by TaMFP1 and TaMFP2 (16.30%). In contrast, potassium content was similar among Th-CP and the control but was negatively affected by the TaMFP1 treatment, leading to a 9.98% reduction.

In the case of micronutrients, no significant improvements were observed in the zinc and iron content with the fungal treatments, as they showed values similar to the control. In contrast, the *Trichoderma* treatment reduced manganese content by 24.11% to 43.93%. Similarly, copper content decreased by up to 16.77% with the TaMFP1 and TaMFP2 treatments.

All treatments exhibited differences in macro- and micronutrient concentrations compared to the optimal ranges recommended for hydroponic spinach nutrition. Nitrogen and potassium macronutrients were within the recommended sufficiency range in all treatments. In contrast, phosphorus, calcium, and magnesium levels were above the sufficiency ranges in all fungal treatments. Regarding micronutrient content, all treatments exhibited nutrient levels within the optimal range. However, copper levels in all treatments exceeded the established recommendations.

### 3.4. Yield of Spinach

**Figure 1:**
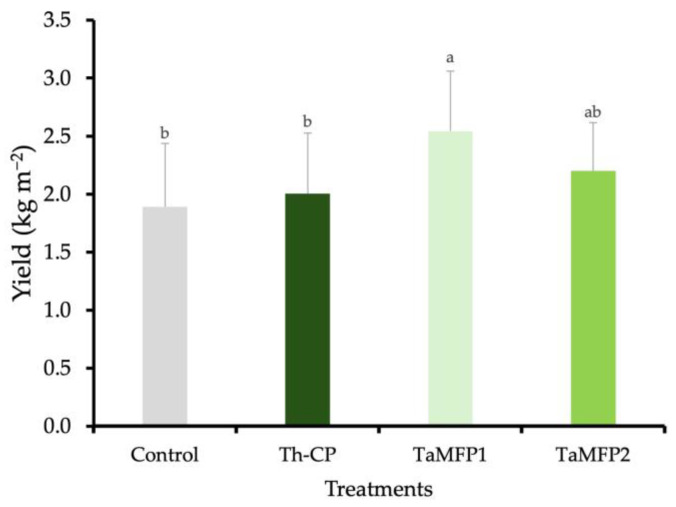
Effect of *Trichoderma* spp. on the yield of spinach cv. Stella Plus F-1 in a Deep-Water Culture system under greenhouse conditions. The control group consists of non-inoculated plants, while Th-CP refers to a commercial formulation containing *Trichoderma harzianum* (Trichospore^®^). Additionally, TaMFP1 and TaMFP2 correspond to *Trichoderma asperellum* strains. Bars sharing the same letter do not exhibit statistically significant differences based on the Tukey test (*p* < 0.05).

The spinach yield was similar among the TaMFP2, Th-CP, and control treatments. The application of TaMFP1 led to the highest spinach yield, showing a 34.46% increase relative to non-inoculated plants. This treatment resulted in an average yield of 2.54 kg m^−2^ (Figure 1).

### 3.5. Visual Quality of Spinach

**Figure 2:**
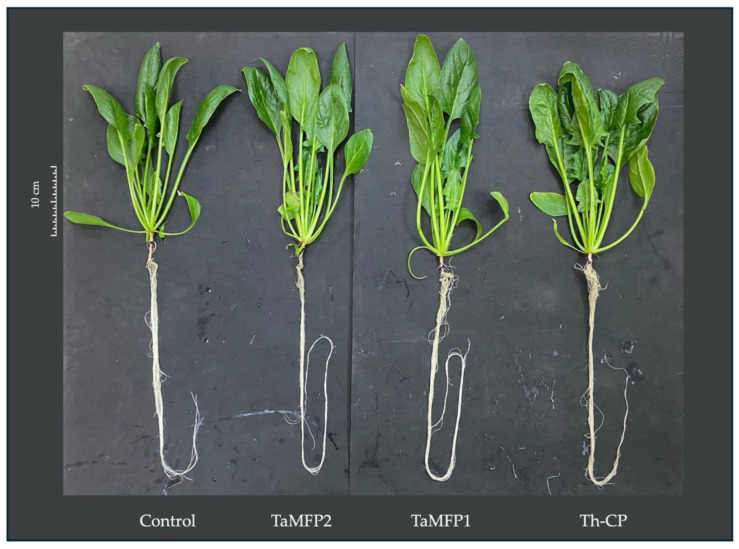
Spinach cv. Stella Plus F-1 treated with *Trichoderma* spp. under greenhouse conditions at 28 days after the first inoculation. Control = non-inoculated plants; TaMFP1 and TaMFP2 = *Trichoderma asperellum*; Th-CP = commercial product based on *Trichoderma harzianum* (Trichospore^®^).

**Figure 3:**
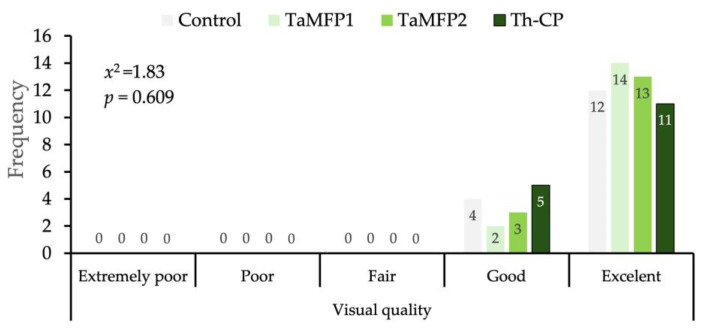
Distribution of visual quality of spinach cv. Stella Plus F-1 treated with *Trichoderma* spp. under greenhouse conditions in a Deep-Water Culture system at 28 days after the first inoculation. Control = non-inoculated plants; TaMFP1 and TaMFP2 = *Trichoderma asperellum*; Th-CP = commercial product based on *Trichoderma harzianum* (Trichospore^®^).

The morphological development of the spinach treated with *Trichoderma* for 28 days in the DWC system was similar to that of the non-inoculated plants without affecting its visual quality (Figure 2). The evaluation of the visual quality of spinach under different treatments showed a homogeneous distribution between the “Good” and “Excellent” categories (Figure 3).

The independence analysis using the Chi-square test did not indicate significant differences between treatments (*x*^2^ = 1.83, *p* = 0.690), indicating that the applied *Trichoderma* treatments did not significantly influence the proportion of spinach classified in each quality category (Figure 3).

### 3.6. Principal Component Analysis

**Figure 4:**
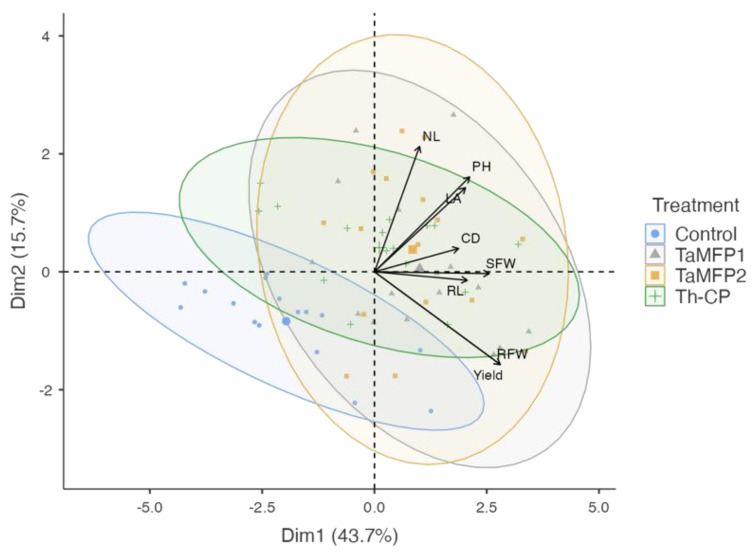
Principal component analysis plot of the growth promotion in spinach plants cv. Stella Plus F-1 treated with *Trichoderma* spp. under greenhouse conditions in a Deep-Water Culture system (KMO 0.755, *X*^2^ = 141, *p* < 0.001). Control = non-inoculated plants; TaMFP1 and TaMFP2 = *Trichoderma asperellum*; Th-CP = commercial product based on *Trichoderma harzianum* (Trichospore^®^). Dim 1 and Dim 2 = principal components; CD = collar diameter; NL = number of leaves; LA = leaf area; RL = root length; SFW = shoot fresh weight; DFW = root fresh weight; PH = plant height.

Principal component analysis allowed for visualization of the variability in the spinach’s response to the different fungi treatments under evaluation (Figure 4). The first principal component (Dim 1) explained 43.7% of the variability, while the second component (Dim 2) accounted for 15.7%, accumulating 59.4% of the total variability.

The treatments with *Trichoderma* (TaMFP1, TaMFP2, and Th-CP) clustered in the positive quadrant of Dim 1, showing a strong association with growth and yield-related variables, such as the number of leaves, plant height, collar diameter, fresh shoot and root biomass, and yield. In contrast, the control group was in the negative quadrant of Dim 1, indicating a lower correlation with growth variables. These results suggest that the application of *Trichoderma* promotes spinach plant development, enhancing vegetative growth and biomass accumulation compared to the control.

## 4. Discussion

This study’s findings demonstrate that inoculating the roots with *Trichoderma asperellum* in a Deep-Water Culture system notably improves the growth and development of Stella F-1 spinach when cultivated under greenhouse conditions. These findings are particularly relevant because although the application of *Trichoderma* in hydroponic crops has been explored in lettuce, arugula, and basil, this is the first study to evaluate its effect on spinach under a Deep-Water Culture system, applied as a spray to the root system. Spinach exhibits a distinct physiological behavior compared to other leafy vegetables, making these results valuable for expanding knowledge on plant–microorganism interactions in this hydroponic system.

These findings align with previous studies on the other leafy crops grown in hydroponic systems and inoculated with different *Trichoderma* species. The improvements observed in vegetative parameters, such as plant height, leaf area, root length, number of leaves, and biomass, are consistent with previous reports. For instance, Gutiérrez-Chávez et al. [14] reported that the same strains evaluated (*T. asperellum* TaMP1 and TaMFP2) promoted lettuce growth, increasing plant height by 19.01%, root length by 25.69%, leaf area by 33.60%, number of leaves by 18.18%, total fresh biomass by 76.40%, and dry biomass by 82.63%. Similarly, Pineda-Acosta et al. [46] also observed that applying *T. harzianum* in a floating root system led to an 11.0% rise in leaf count and a 63.0% enhancement in foliar biomass in lettuce. Leu et al. [47] recorded a 95.4% increase in the height of basil (*Ocimum basilicum* L.) grown in a floating root hydroponic system after the application of *T. atroviride* αCP8.

Consistent results have been observed in other hydroponic systems. Promwee and Intana [12] demonstrated that *T. asperellum* NST-009 promoted the growth of Green Oak lettuce in a commercial nutrient film technique (NFT) hydroponic system, with increases of 8.62% in plant height, 18.39% in the number of leaves, 25.71% in fresh aerial biomass, and 39.26% in fresh root biomass. Moreira et al. [41] recorded a 20.0% increase in root length in lettuce grown using an NFT after foliar inoculation with *T. harzianum* ESALQ-1306. Oliveira et al. [48] observed a significant increase in arugula (*Eruca vesicaria* L.), with a 24.4% rise in fresh aerial biomass and 74.5% and 544% increases in fresh and dry root biomass, respectively, following inoculation with *T. harzianum* in an NFT system. Moreover, Yedidia et al. [49] reported that inoculation with *Trichoderma* sp. strain T-203 increased the number of leaves in cucumber by 3.2 times in an axenic hydroponic system.

The enhancement of spinach growth following *Trichoderma* application could be linked to its capacity to promote the formation of primary meristematic tissues and improve root system architecture. Specifically, *Trichoderma* has been reported to increase root hair density and elongation, thereby improving water and nutrient absorption efficiency [50,51]. This enhanced root development could explain the significant growth response observed in spinach in this study. Furthermore, *Trichoderma* influences root system architecture by modulating auxin signaling, as reported by Garnica-Vergara et al. [52], which promotes lateral root formation and overall root system expansion. These findings suggest that *Trichoderma* may enhance nutrient uptake by optimizing root morphology and increasing root surface area. A more extensive root system facilitates increased nutrient and water acquisition, ultimately supporting overall plant growth. Additionally, *Trichoderma*-mediated rhizosphere colonization has been linked to physiological enhancements beyond root development. Vargas et al. [53] reported that *Trichoderma virens* can improve photosynthetic efficiency and CO_2_ assimilation in maize plants (*Zea mays* L.), contributing to higher biomass accumulation and overall plant vigor.

The increase in biomass observed in this study is likely a result of enhanced nutrient uptake efficiency, which can be attributed to *Trichoderma*’s capacity to produce secondary metabolites and hydrolytic enzymes that improve root health and nutrient assimilation. Previous studies have demonstrated that *Trichoderma* species produce compounds such as indole-3-acetic acid, siderophores, and lytic enzymes that contribute to plant growth and stress tolerance [54,55,56].

In this study, no significant differences were found in the contents of chlorophyll *a*, chlorophyll *b*, and carotenoids between treatments. This result is similar to that reported by Pineda-Acosta et al. [46], who found that applying *T. harzianum* in lettuce grown in a floating root system did not significantly alter the content of photosynthetic pigments. Similarly, Gutiérrez-Chávez et al. [14] found no effect of *T. harzianum* and *T. asperellum* (TaMFP1 and TaMFP2) on the content of photosynthetic pigments in lettuce grown in a floating root system. The stability of pigments suggests that the impact of *Trichoderma* on spinach growth occurs mainly through improved nutrient absorption rather than a direct increase in photosynthetic activity. However, it remains unclear whether this stability is due to an actual lack of effect or a compensatory physiological response of spinach to maintain chlorophyll homeostasis. Additional studies measuring photosynthetic efficiency and gas exchange parameters would be necessary to confirm this hypothesis.

The results obtained in this study not only confirm but also provide a broader understanding of previous findings on the influence of *Trichoderma* spp. on nutrient absorption and assimilation in hydroponic crops. In particular, the significant increase in nitrogen and calcium levels in spinach treated with *T. harzianum* (Th-CP) is consistent with the findings of Oliverira et al. [48] and Caruso et al. [57], who reported increases of 51.77% and 19.2%, respectively, in the absorption of these elements in hydroponic arugula following the application of *T. harzianum*. This enhancement in nutrient uptake can be attributed to the ability of *Trichoderma* spp. to modify root architecture and promote the expression of genes related to nutrient transport and assimilation, as demonstrated by Meng et al. [58] in cucumber crops. On the other hand, the increase in phosphorus content with the TaMFP1 treatment also supports the results of Caruso et al. [57], who observed an 18.2% increase in phosphate adsorption in arugula treated with *T. harzianum* T22. The mechanism behind phosphorus solubilization by *Trichoderma* could be due to the secretion of low-molecular-weight organic acids that enhance the bioavailability of this nutrient in the rhizosphere, improving its uptake and translocation to plant tissues, as noted by Bonini et al. [59].

However, in contrast to the studies by Rouphael et al. [60], who reported a significant increase in potassium absorption in lettuce after the application of *T. virens*, in our study, potassium levels remained stable between the Th-CP and TaMFP2 treatments, while a 9.98% reduction was observed with TaMFP1. This discrepancy may be explained by a specific interaction between the strain used and the availability of K in the nutrient solution, which could affect ionic regulation in the plant.

Regarding micronutrients, a significant decrease in manganese and copper concentrations was observed with the TaMFP1 and TaMFP2 treatments. This finding is consistent with the report by de Santiago et al. [61], who indicated that *T. asperellum* competes with plants to absorb copper, manganese, and zinc, which could explain the reduction in these elements in spinach. Despite this decrease, the levels of all micronutrients remained within the sufficiency range, suggesting that *Trichoderma* inoculation did not cause nutritional deficiencies in the spinach.

The results obtained in this study showed that inoculation with the TaMFP1 strain increased spinach yield. These findings are consistent with those reported by Gutiérrez-Chávez et al. [14], who observed that the application of *T. asperellum* (TaMFP1 and TaMFP2) and *T. harzianum* increased foliar yield by 78.49% in hydroponic lettuce. However, the magnitude of the increase in this study was lower, which could be attributed to differences in the plant species evaluated and the conditions of the hydroponic system. In comparison with other studies, Oliveira et al. [48] reported that the inoculation of *T. harzianum* in a hydroponic arugula system significantly improved yield, reaching up to 5.16 kg m^−2^. Meanwhile, Pereira et al. [62] reported up to 66% increases in hydroponic lettuce productivity using the *T. harzianum* ESAQ1306 strain. In this context, although the yield increase in spinach with TaMFP1 in this study is significant, its impact is lower than that observed in crops such as arugula and lettuce, which could be due to differences in species physiology and their response to symbiosis with *Trichoderma*.

The positive effect of *Trichoderma* on hydroponic crop yield can be attributed to physiological and biochemical mechanisms. Oliveria et al. [48] and Pereira et al. [62] suggest that *T. harzianum* enhances root structure and optimizes nutrient adsorption, allowing better assimilation of available mineral resources. Additionally, *Trichoderma*’s ability to stimulate the synthesis of phytohormones could explain the increase in plant biomass observed in this study.

The morphological development of spinach treated with *Trichoderma* in the Deep-Water Culture system at 28 days was similar to that of non-inoculated plants without affecting its visual quality. These results are consistent with those reported by Gutiérrez-Chávez et al. [14], who found that the visual quality of hydroponically grown lettuce showed no statistically significant differences between lettuce inoculated with *T. asperellum* (TaMFP1 and TaMFP2) and non-inoculated ones. This behavior suggests that, under controlled hydroponic conditions, the application of *Trichoderma* does not compromise the visual appearance of leafy vegetables.

From an agronomic perspective, these results indicate that inoculation with *T. asperellum* may be a viable strategy to increase spinach yield in hydroponic systems without altering its quality. This finding is crucial for commercial production, as the visual appearance of spinach is a key marketing criterion. Additionally, the improvement in nutrient absorption suggests a potential reduction in fertilizer use, which could contribute to the sustainability of the production system. However, further research is needed to assess its effect on different crops and varieties, optimize its application, and better understand its mechanisms of action in soilless systems. It is essential to analyze its interaction with the nutrient solution and stability under commercial conditions to maximize efficiency. Additionally, studying its impact on postharvest quality and its compatibility with other beneficial microorganisms will help develop more sustainable strategies for hydroponic vegetable production.

## 5. Conclusions

This study confirms that inoculation with *T. asperellum* significantly enhances the growth and development of spinach cultivated in a Deep-Water culture system. Treatments with *T. asperellum* (TaMFP1 and TaMFP2) increased total biomass, leaf number, and root length without affecting the product’s visual quality. Additionally, an increase in the absorption of certain macronutrients, particularly phosphorus and calcium, was observed, although variations were noted in the levels of some micronutrients, such as manganese and copper.

The findings suggest that *T. asperellum* has potential as a biostimulant in hydroponic crops, offering a sustainable alternative to reduce reliance on chemical fertilizers without compromising productivity or product quality. However, a deeper understanding of the interaction mechanism between *T. asperellum* and the rhizosphere under soilless cultivation conditions is required to optimize its application and ensure its efficacy across different environmental conditions and plant species.

These results contribute to developing beneficial microorganism-based strategies to enhance the sustainability of hydroponic agriculture, opening new perspectives for integrating bio-inputs into the commercial production of high-value vegetables.

## Figures and Tables

**Table 1:** Assessment scale for spinach quality through visual product analysis [42].

Score	Description	Visual Quality
9	Defect-free, freshly harvested.	Excellent
7	Some leaves slightly yellowed or decayed or slight loss of turgor or some physical damage (<10%).	Good
5	Fair, moderately objectionable defects, limit of marketability.	Fair
3	Most leaves yellowed or decayed and considerable loss of turgor.	Poor
1	The product is entirely unfit for use, exhibiting complete chlorosis, mold growth, an unpleasant odor, excessive root development, and visible signs of physical deterioration.	Extremely poor

**Table 2:** Effect of *Trichoderma* spp. on the growth of spinach cv. Stella Plus F-1 in a Deep-Water Culture system under greenhouse conditions at 28 days after first inoculation.

Parameters ^1^	Treatments			
	Control	Th-CP	TaMFP1	TaMFP2
Plant height (cm)	25.11 ± 2.78 ^b^	31.73 ± 1.66 ^a^	30.46 ± 2.84 ^a^	30.54 ± 2.01 ^a^
Collar diameter (cm)	4.00 ± 0.057 ^b^	4.69 ± 0.75 ^a^	5.08 ± 0.70 ^a^	4.83 ± 0.58 ^a^
Root length (cm)	52.62 ± 7.55 ^b^	74.02 ± 14.25 ^a^	68.39 ± 19.01 ^a^	78.01 ± 1.74 ^a^
Leaf area (cm^2^ plant^−1^)	453.59 ± 84.71 ^b^	543.43 ± 94.04 ^a^	559.94 ± 64.14 ^a^	556.87 ± 121.71 ^a^
Number of leaves (plant^−1^)	13.13 ± 1.50 ^b^	14.00 ± 1.97 ^ab^	15.56 ± 2.00 ^a^	15.44 ± 2.78 ^a^
Leaf area ratio (cm^2^ g^−1^) *	261.02 ± 51.38 ^a^	248.63 ± 48.36 ^a^	260.98 ± 38.73 ^a^	248.96 ± 65.27 ^a^
Specific root length (m g^−1^) **	2.85 ± 0.63 ^b^	3.27 ± 0.63 ^ab^	3.12 ± 1.27 ^ab^	3.79 ± 0.97 ^a^

^1^ The results are expressed as means ± standard deviation from four plants per replicate, with a total of four replicates. Distinct superscript letters within the same row denote significant differences based on the Tukey test, Games–Howell test *, or Conover–Iman test ** at a significance level of 0.05. The control group consists of non-inoculated plants, while Th-CP refers to a commercial formulation containing *Trichoderma harzianum* (Trichospore^®^). Additionally, TaMFP1 and TaMFP2 correspond to *Trichoderma asperellum* strains.

**Table 3:** *Trichoderma* spp. effect on the biomass of spinach cv. Stella Plus F-1 in a Deep-Water Culture system under greenhouse conditions at 28 days after first inoculation.

Parameters ^1^	Treatments			
	Control	Th-CP	TaMFP1	TaMFP2
Fresh biomass (g plant^−1^)				
Leaves	24.54 ± 4.94 ^b^	28.00 ± 5.54 ^ab^	28.75 ± 4.01 ^ab^	31.64 ± 6.02 ^a^
Root	5.56 ± 1.60 ^b^	5.89 ± 1.54 ^b^	7.48 ± 1.52 ^a^	6.47 ± 1.22 ^ab^
Total	30.11 ± 6.23 ^b^	33.89 ± 6.66 ^ab^	36.23 ± 4.45 ^a^	38.11 ± 6.80 ^a^
Dry biomass (g plant^−1^)				
Leaves	1.57 ± 0.28 ^b^	2.00 ± 0.31 ^a^	1.93 ± 0.21 ^a^	2.07 ± 0.36 ^a^
Root	0.19 ± 0.3 ^b^	0.23 ± 0.05 ^ab^	0.24 ± 0.05 ^a^	0.21 ± 0.04 ^ab^
Total	1.76 ± 0.30 ^b^	2.22 ± 0.35 ^a^	2.16 ± 0.20 ^a^	2.28 ± 0.39 ^a^
Shoot–root ratio (g g^−1^)	8.41 ± 1.36 ^a^	8.81 ± 1.28 ^a^	8.70 ± 2.70 ^a^	9.86 ± 1.45 ^a^

^1^ The results are expressed as means ± standard deviation from four plants per replicate, with a total of four replicates. Distinct superscript letters within the same row denote significant differences based on the Tukey test at a significance level of 0.05. The control group consists of non-inoculated plants, while Th-CP refers to a commercial formulation containing *Trichoderma harzianum* (Trichospore^®^). Additionally, TaMFP1 and TaMFP2 correspond to *Trichoderma asperellum* strains.

**Table 4:** *Trichoderma* spp. effect on photosynthetic pigments of spinach cv. Stella Plus F-1 in a Deep-Water Culture system under greenhouse conditions at 28 days after first inoculation.

Parameters ^1^		Treatments		
	Control	Th-CP	TaMFP1	TaMFP2
Photosynthetic pigments (mg g^−1^ FW)				
Chlorophyll a **	1.25 ± 0.056 ^a^	1.26 ± 0.32 ^a^	1.26 ± 0.030 ^a^	1.23 ± 0.085 ^a^
Chlorophyll b *	0.75 ± 0.245 ^a^	0.60 ± 0.108 ^a^	0.54 ± 0.082 ^a^	0.57 ± 0.149 ^a^
Carotenoids	0.574 ± 0.080 ^a^	0.534 ± 0.040 ^a^	0.527 ± 0.58 ^a^	0.532 ± 0.057 ^a^

^1^ The results are expressed as means ± standard deviation from four plants per replicate, with a total of four replicates. Distinct superscript letters within the same row denote significant differences based on the Tukey test, Games–Howell test *, or Conover–Iman test ** at a significance level of 0.05. The control group consists of non-inoculated plants, while Th-CP refers to a commercial formulation containing *Trichoderma harzianum* (Trichospore^®^). Additionally, TaMFP1 and TaMFP2 correspond to *Trichoderma asperellum* strains.

**Table 5:** *Trichoderma* spp. effect on the macro and micronutrients of spinach cv. Stella Plus F-1 in a Deep-Water Culture system under greenhouse conditions at 28 days after first inoculation.

Parameters ^1^			Treatments		Sufficiency Range ^2^
	Control	Th-CP	TaMFP1	TaMFP2	
Macronutrients (%)					
N *	4.58 ± 0.31 ^b^	5.44 ± 0.37 ^a^	5.30 ± 0.54 ^ab^	4.50 ± 0.27 ^b^	4.0–6.0
P	1.12 ± 0.08 ^b^	1.20 ± 0.09 ^ab^	1.27 ± 0.04 ^a^	1.20 ± 0.07 ^ab^	0.3–0.5
K	4.31 ± 0.09 ^ab^	4.50 ± 0.13 ^a^	3.88 ± 0.15 ^c^	4.09 ± 0.23 ^bc^	3.0–8.0
Ca	1.35 ± 0.11 ^c^	1.79 ± 0.06 ^a^	1.60 ± 0.07 ^b^	1.54 ± 0.04 ^b^	1.0–1.5
Mg	1.64 ± 0.04 ^b^	1.72 ± 0.03 ^a^	1.69 ± 0.02 ^ab^	1.69 ± 0.01 ^ab^	0.4–1.0
Micronutrients (ppm)					
Fe *	102.75 ± 19.36 ^a^	93.13 ± 2.96 ^a^	90.25 ± 7.85 ^a^	82.63 ± 1.38 ^a^	50–200
Mn *	74.10 ± 9.78 ^a^	53.43 ± 5.84 ^bc^	41.55 ± 1.78 ^c^	56.23 ± 3.27 ^b^	25–200
Cu *	24.63 ± 2.32 ^a^	23.25 ± 2.22 ^ab^	21.00 ± 0.41 ^b^	20.00 ± 1.08 ^b^	5–15
Zn	32.63 ± 4.99 ^ab^	29.00 ± 5.28 ^b^	37.63 ± 4.32 ^ab^	41.35 ± 2.91 ^a^	20–75

^1^ The results are expressed as means ± standard deviation from four plants per replicate, with a total of four replicates. Distinct superscript letters within the same row denote significant differences based on the Tukey test or Games–Howell test * at a significance level of 0.05. The control group consists of non-inoculated plants, while Th-CP refers to a commercial formulation containing *Trichoderma harzianum* (Trichospore^®^). Additionally, TaMFP1 and TaMFP2 correspond to *Trichoderma asperellum* strains. ^2^ Values corresponding to the growth of hydroponic spinach, according to Campbell [45].

## Data Availability

The data presented in this study are available upon request from the corresponding authors.
